# Turning Meadow Weeds Into Valuable Species for the Romanian Ethnomedicine While Complying With the Environmentally Friendly Farming Requirements of the European Union’s Common Agricultural Policy

**DOI:** 10.3389/fphar.2020.00529

**Published:** 2020-04-23

**Authors:** Elena Grosu, Mihael Cristin Ichim

**Affiliations:** “Stejarul” Research Centre for Biological Sciences, National Institute of Research and Development for Biological Sciences, Piatra Neamt, Romania

**Keywords:** meadow weed, medicinal plant, ethnomedicine, *Arctium lappa*, *Eryngium campestre*, *Rumex acetosella*, *Xanthium spinosum*, *Xanthium strumarium*

## Abstract

The cross-compliance mechanism of the European Union (EU)'s common agricultural policy (CAP) makes the approval of the direct payments to the European farmers subject to compliance with the requirement to maintain the land in good agricultural and environmental condition. One of the obligations of the Romanian land owners and farmers is to avoid the installation of unwanted vegetation on their land plots. This vegetation is represented by some species of herbaceous or woody plants, annual or perennial, that spontaneously invade the agricultural lands, diminishing the production capacity of the cultivated plants. Included in this category are 10 meadow weeds, without fodder value or even toxic to animals: *Arctium lappa* L., *Carduus nutan*s L., *Conium maculatum* L., *Eryngium campestre* L., *Euphorbia cyparissias* L., *Pteridium aquilinum* (L.) Kuhn, *Rumex acetosella* L., *Veratrum album* L., *Xanthium spinosum* L., and *Xanthium strumarium* L. Various and multiple uses in traditional medicine of these meadow weed species have been reported for Romania and other nine neighboring East European countries, *i.e.* Bosnia and Herzegovina, Bulgaria, Czech Republic, Estonia, Kosovo, Russia, Turkey, Serbia, and Ukraine. For *A. lappa* were recorded the highest number of ethnomedicinal uses, in the largest number of East European countries, including Romania. *C. maculatum* and *V. album* are not recommended for human consumption but can be further investigated as potential sources of pharmaceutically active compounds. Once removed by landowners and farmers from their land, the raw plant material of these 10 species become readily and easily available to the Romanian local communities and the industry of herbal food supplements, while the biodiversity of the agro-ecosystems is maintained.

## Introduction

The European Union (EU)'s common agricultural policy (CAP) is one of the world's largest agricultural policies and the EU's longest-prevailing one (Pe'er et al., 2019). Besides producing food and stimulating rural community development, the CAP defines one more condition that allows farmers to fulfill their functions in society—environmentally sustainable farming—to produce food while simultaneously protecting nature and safeguarding biodiversity (European Commission, 2020a). Cross-compliance is a CAP's mechanism that links direct payments to compliance by farmers with basic standards concerning the environment, food safety, animal and plant health and animal welfare, as well as the requirement of maintaining land in good agricultural and environmental condition (GAEC). Evidence has shown that agriculture is the single largest cause of biodiversity loss but already nearly one-third of the world's farms have adopted more environmentally friendly practices while continuing to be productive (Pretty et al., 2018). In Europe, since 2005, all EU farmers receiving direct payments under CAP are subject to compulsory cross-compliance which is an important tool for integrating environmental requirements into the CAP (European Commission, 2020b). The good GAECs and the statutory management requirements (SMR), refer to a set of EU standards and requirements aiming at sustainable agriculture (European Parliament, 2013). In the area “Environment, climate change, good agricultural condition of land,” when referring to the issue of “Landscape, minimum level of maintenance,” GAEC 7 requires “Retention of landscape features, including where appropriate, hedges, ponds, ditches, trees in line, in group or isolated, field margins and terraces, and including a ban on cutting hedges and trees during the bird breeding and rearing season and, as an option, measures for avoiding invasive plant species” (European Parliament, 2013). After they are defined and detailed at national or regional level, all these standards are to be respected by all the European farmers receiving direct payments or some of the rural development payments.

## Methods: Literature Search Strategy

We systematically searched four databases (Web of Science, PubMed, Scopus, and ScienceDirect) for relevant, peer reviewed publications, using a combination of relevant keywords and Boolean operators which included the name of the 10 plant species and of the Eastern European countries, with no other filters applied (in July 2019). The number of retrieved abstracts was below 10, irrespective of the species searched for. This result was somehow anticipated by the guest editors of the research topic when they expected that “ethnopharmacological research in this area is quite limited, many of the existing studies being published in national or local journals, thus being less visible to the scientific community.” To expand the literature search, Google Scholar database was interrogated, and all the retrieved publication was selected based in the information provided by their abstracts and subsequently by their full text version. Only peer-reviewed articles were included in our review. To access the literature published in the Romanian language, the online catalog of the County Library “G. T. Kirileanu” Neamt was searched using the eBibliophil search engine (https://bibgtkneamt.ebibliophil.ro). All the relevant specialty books retrieved were searched for the 10 plant species and if they contain any recommended use in ethnomedicine.

Selection criteria: all the publications referring to the 10 plant species and their use in the ethnomedicine of any of the Eastern European countries (from the former communist countries in the west till Russia, Ukraine and Turkey in the east) have been included in our literature review.

## Romania's Provisions for Avoiding the Installation of Unwanted Vegetation on the Farming Land

Romania covers an area of 238,391 km² of which around 61% agricultural land (14.6 million ha) (64.2% arable land, 32.9% meadows and natural grasslands and 2.7% plantations of trees and vineyard), and 28.3% forests and other forestry vegetation lands. The Rural Development Programme (RDP) is outlining Romania's priorities for using the nearly € 9.5 billion of public money that is available for the CAP 2014–2020 (European Commission, 2015).

In agreement with the EU's regulations, Romania has defined three measures to be followed in order to implement the GAEC 7, one of which imposes that farmers have to prevent unwanted vegetation to install on the farming land, including on the uncultivated one. The Agency for Payments and Intervention in Agriculture (APIA) is the national authority responsible for coordinating the control activity on the cross-compliance norms within the schemes and support measures for the Romanian farmers (Ministry of Agriculture and Rural Development, 2015).

The details regarding the application of the measures of GAEC 7 have been modified along the years but its main objective was to maintain a minimum level of maintenance of agricultural land (regardless of the category of use, including land which is no longer used for production) by avoiding the installation of unwanted vegetation. In this context, the unwanted vegetation is represented by some species of herbaceous or woody plants, annual or perennial, that spontaneously invade the agricultural lands, diminishing the production capacity of the cultivated plants. Also included in this category are some meadow plant species, without fodder value or even toxic to animals.

The legal provisions recommend that the unwanted vegetation should not dominate the culture in more than 30% of the plot area, regardless of the land use category (arable land, permanent meadow, or permanent crops). However, when the percentage of weediness exceeds the threshold of 80% of the surface of the plot, the entire plot is considered ineligible and excluded from payment. Also, the uncultivated agricultural land, one or more years, on which the unwanted vegetation was installed on more than 80% of the area, is excluded from payment. This decision is taken after on-site inspections carried out by APIA representatives (Agency for Payments and Intervention in Agriculture, 2019b).

## The Unwanted Meadow Weeds in Romania

The main weed species that make up the unwanted vegetation on meadows (plants without fodder value or toxic plants) are annual, biennial or perennial plants. The APIA has listed 10 biannual or perennial weeds, all to be removed by the Romanian land owners or farmers from the meadows for which direct payments (EU subsidies) are requested: *Arctium lappa* L., *Carduus nutans* L., *Conium maculatum* L., *Eryngium campestre* L., *Euphorbia cyparissias* L., *Pteridium aquilinum* (L.) Kuhn, *Rumex acetosella* L., *Veratrum album* L., *Xanthium spinosum* L., and *Xanthium strumarium* L. (Agency for Payments and Intervention in Agriculture, 2019b). The same regulating and control body has prepared and publicly released a guide for identifying these 10 species that represent unwanted vegetation growing on the meadows (Agency for Payments and Intervention in Agriculture, 2019a). For each of the 10 species, a detailed description of the plant (roots, stem, leaves, flowers, and seeds), seed dispersal mechanism, and the general ecology of the species is provided, visually supported by four to six pictures. All these details are meant to facilitate the on-the-field identification for removal by the interested individuals.

To achieve its final goal, *i.e.* the removal of these 10 species from the EU-subsidized fields, APIA should simultaneously act by stopping and reversing the continuous degradation of grasslands due to overgrazing, a consequence of the larger, and increasing, number of grazing animals, also receiving a per-capita EU subsidy from the same governmental body: APIA. Overgrazing leads to the invasion of ruderal plant species, later on categorized as “unwanted” weed species by APIA. From the phytosociological point of view, two of the 10 species, *i.e. E. cyparissias* and *R. acetosella*, are natural components of most of the grasslands in the vegetation classes Festuco–Brometea (*E. cyparissias*) and Molinio–Arrhenatheretea (*R. acetosella*) (Sanda et al., 2008; Chifu, 2014), and they were erroneously categorized as unwanted vegetation in APIA's guide (Agency for Payments and Intervention in Agriculture, 2019a). Moreover, some of the other 10 species are not the most common and widespread species of their genus in Romania, *e.g*. *Carduus acanthoides* invades many more hectares of degraded grasslands than *C. nutans* (Sarateanu et al., 2008). All the above considered, the APIA's documents with respect to the invasive and unwanted vegetation should be critically reviewed and updated for the future post-2020 CAP.

## Phytochemical Constituents and Ethnomedicinal Uses of the Meadow Weeds

All 10 species have ethnomedicinal uses which were reported around the world, including in Romania and other neighboring Eastern European countries, some of them dating back for centuries or even millennia. Their traditional use as edible and medicinal species has triggered investigations on their phytochemical composition.

### *A. lappa* L.—Greater Burdock, *Asteraceae*

*A. lappa* has been used therapeutically in Europe, North America and Asia for hundreds of years (Wang et al., 2019). *A. lappa* is used in Eastern European folk medicine as an adjuvant in diabetes therapy (European Medicine Agency, 2011; Tousch et al., 2014; Tabassum et al., 2019), to treat digestive, renal, lung, and skin affections, having also depurative, diuretic, and diaphoretic properties (European Medicine Agency, 2011). Traditionally, the roots, buds, and seeds serve as blood purifier, and have been used as remedy for rheumatism, scurvy, gravel, venereal disease, and sores (Di Novella et al., 2013). An improvement of oxidative stress and inflammatory status was observed in patients with knee osteoarthritis treated with *A. lappa* root tea (Maghsoumi-Norouzabad et al., 2016), as well as in treating inflammatory acne (Miglani and Manchanda, 2014). In Greece, *A. lappa*'s root is used for musculoskeletal diseases, in particular against joint pains and rheumatism (Tsioutsiou et al., 2019). In Bosnia and Herzegovina this plant is used to treat skin conditions, to strengthen the hair root, against intestinal parasites, for dissolution of kidney stone, for improved urination, against diabetes and rabies, dog's bites, to release stomach gasses, against sexually transmitted diseases (STDs), and to treat facial nerve inflammation etc. (Redzic, 2010).

The main bioactive compounds identified in all parts of *A. lappa* are the lignans (arctigenin, artiin, matairesinol) and polyphenolic acids (caffeic acid derivatives) (Ferracane et al., 2010; da Silva et al., 2013; Edwards et al., 2015). These two groups possess high antioxidant activity (Liu et al., 2012). Arctigenin is reducing the inflammatory process through inhibiting iNOS expression and promoting cytokines (TNF-α and IL-6) productions (Zhao et al., 2009). *In vitro* studies show that lignans extracted from *A. lappa* have anti-proliferative effect against cancerous cells, inducing apoptosis and limiting migration of metastasis (Lou et al., 2017; Wang et al., 2019).

Several studies showed that the seeds are rich in caffeic acid, chlorogenic acid and cynarin, the leaves contain high amounts of phenolic acids, quercetin, quercitrin, luteolin, sesquiterpenes, and eudesmol, while the roots are rich in carbohydrates (including inulin up to 45%–50%, mucilage, pectin and sugars), arctic acid, polyacetylenes, arctiin, luteolin, quercetin rhamnoside (Ferracane et al., 2010; European Medicine Agency, 2011; Edwards et al., 2015), and caffeoylquinic acids derivatives (Tousch et al., 2014).

In Romanian ethnomedicine *A. lappa* is recommended for its detoxifying (due to the high fiber composition), hepatoprotective (due to caffeic acid derivatives), cholesterol lowering (reduces the absorption of cholesterol and lipids at intestine level), diuretic, antiinflammatory, antioxidative (due to caffeic acid derivatives), hypoglycemic (due to high innulin content) (Bojor and Pop, 2010), and antibiotic effects (Stanescu et al., 2014). This plant is recommended for hepatobiliary and liver protection, hypercholesterolemia, dermatitis, eczema, skin infection, hyperglycemia, cholelithiasis, oily and seborrheic skin, hair regrowth (Bojor and Alexan, 1984; Parvu, 2000; Ardelean et al., 2008; Mohan, 2008; Bojor and Pop, 2010), intoxications, flu, high blood pressure, hemorrhages (Parvu, 2000; Mohan, 2008), muscular atrophy, gout, enterocolitis (Mohan, 2008), herpes, dandruff (Parvu, 2000; Ardelean et al., 2008), cystitis (Bojor and Alexan, 1984), burns (Bojor, 2018), hepatitis, bronchitis, scurvy (Milica et al., 2012), corns, typhoid fever, heart pain, chest pain, rheumatism, STDs, skin rashes (Stanescu et al., 2014), epilepsy, arthritis, constipation, abdominal colic, hepatitis, and insect bites (Grigore and Grigore, 2007).

### *C. nutans* L. —Musk Thistle, Asteraceae

*C. nutans* is used as dietary supplement (Isik and Yucel, 2017). In Turkish folk medicine, this species has been used as tonic to stimulate liver function, detoxifying herb and to ameliorate fever (Aktay et al., 2000). In Bulgaria, *C. nutans* is used for its antihemorroidal, cardiotonic and diuretic properties (Zheleva-Dimitrova et al., 2011).

The main biochemical compounds of musk thistle are the flavonoids (apigenin, luteolin, kempferol and derivatives, rutin, tilianin, and isorhamnetin), sterols and triterpenes (β-sitosterol acetate, sitosterol-O-xyloside, taraxasterol acetate), polyacetylenes (vinylpentacetylene), phenolic acids, and anthocyanins (Bain and Desrochers, 1988; Abdallah and Ramadan, 1989; Jordon-Thaden and Louda, 2003; Zhelev et al., 2013). Flavonoids represents an important class of compounds known to have a positive impact in ameliorating symptoms of cancer, cardiovascular disease, and neurodegenerative disorders (Spencer et al., 2004; Salvamani et al., 2014). In the last decade, studies on apigenin use as an adjuvant in cancer treatment had shown tremendous results, this plant originated flavone acting as a chemoprotective agent, inhibiting tumor development through inducing cycle arrest and apoptosis (Banerjee and Mandal, 2015; Kashyap et al., 2018).

In Romanian traditional medicine (TM) the *C. nutans*'s seeds are used to prevent atherosclerosis, the flowers as febrifuge, blood purifier (Daraban et al., 2013) and used for treating polyarticular ankilosis, and myalgia, while the leaves are recommended for hypertension and liver diseases (Grigore and Grigore, 2007).

### *C. maculatum* L.—Poison Hemlock, *Apiaceae*

*C. maculatum* is one of the most poisonous plants for laboratory animals, farm animals, and human beings, due to the presence of piperidine alkaloids in all its parts (Al-Snafi, 2016). Despite its poisonous nature, *C. maculatum* is included in several herbals as *Succus conii*, described as a narcotic, sedative, analgesic, spasmolytic, anti-aphrodisiac, and anti-cancer agent (Reynolds, 2005). *C. maculatum* extract is used as a traditional homeopathic remedy for cervix carcinoma (Mondal et al., 2014). This species has been used in ethnomedicine as an analgesic and anti-inflammatory agent (De Landoni and Conium maculatum, 1990; Arihan et al., 2009; Al-Snafi, 2016,; Madaan and Kumar, 2012), in Turkey to treat diabetes (Paksoy et al., 2016), and in Morocco as an alternative to treat typhoid fever and sterility, and also to ease labor (Kharchoufa et al., 2018). Externally, it has been used to treat herpes and swelled joints (Bloch, 2001). Some studies highlight the antispasmodic property of *C. maculatum* and it was reported to have a positive impact on patients with epilepsy, asthma, angina, rheumatism, and tetanus (Mitich, 1998; Hotti and Rischer, 2017).

The chemical composition of poison hemlock has been widely studied and the main toxic alkaloids are coniine and γ-coniceine identified in all plant parts, but mostly in roots and seeds, and they are also responsible for the sedative and anti-inflammatory properties of the species (Vetter, 2004; Panter et al., 2011; Cortinovis and Caloni, 2015; Al-Snafi, 2016; Kharchoufa et al., 2018). Besides alkaloids, *C. maculatum* is rich in flavonoids (anti-oxidative), coumarins (anti-microbial, anti-inflammatory), polyacetylenes, vitamins, and oils (Al-Snafi, 2016).

In Romanian ethnomedicine, *C. maculatum* was used as sedative (Bojor, 2018) and for reducing neuralgia (Ardelean et al., 2008; Bojor, 2018).

### *E. campestre* L.—Field Eryngo, *Apiaceae*

*E. campestre* has been used in folk medicine (Zhang et al., 2008). In the European herbal medicine this plant was used as an infusion for the treatment of whooping cough, as well as in the treatment of kidney and urinary tract inflammations (Medbouhi et al., 2018). In Eastern European TM field eryngo roots and leaves are used for their anti-inflammatory, antiscorbutic, diaphoretic, antitussive, diuretic, expectorant, appetite-stimulant, and aphrodisiac properties, and to treat hemorrhoids, rheumatism, and infertility (Küpeli et al., 2006; Belda et al., 2013; Güneş et al., 2014; Conea et al., 2015; Kikowska et al., 2016; Soumia, 2018). In Turkey is used to treat intestinal disorders, flatulence, hepatitis, digestion disorders, and muscle pain (Akgul et al., 2018) but is also used fresh for human consumption (Demirci and Özkan, 2014). A study by Hawas et al., 2013 suggests its potential application in the treatment of Alzheimer's disease.

*E. campestre* represents an important source of multi-target antimicrobial essential oils, with spathulenol being the main chemical compound of the species (Erdem et al., 2015). Several studies showed that *E. campestre* is rich in flavonoids (quercetin glycosides, isorhamnetin glycosides, and myricetin glycosides), phenolic acids, acetylenes, saponins, steroids, terpenoids, and coumarins (Abou El-Kassem et al., 2013; Marčetić et al., 2014; Matejić et al., 2018; Soumia, 2018). The flavonoids and phenolic compounds contribute to the antioxidant properties of the plant (Küpeli et al., 2006; Matejić et al., 2018; Soumia, 2018).

In Romanian ethnomedicine *E. campestre* is recommended for its cholagogue, diuretic, appetite-stimulant (Craciun et al., 1976; Grigore and Grigore, 2007), expectorant, antitussive, bronchial antiseptic, antispasmodic, antibiotic, hypotensive, and carminative effects (Milica et al., 2012), and is used for treating gallbladder, urinary retention, gonococcal urethritis, ascites, amenorrhea, scurvy, tuberculosis, acne, oliguria, colicky nephritis, seborrheic dermatitis, leg edema (Grigore and Grigore, 2007), amenorrhea, constipation, tooth pain, periodontitis, tooth decay (Milica et al., 2012), and nephrolithiasis (Ardelean et al., 2008). In the Romanian rural areas is used as ingredient in various dishes, especially in soups (Parvu, 2000).

### *E. cyparissias* L.—Cypress Spurge, *Euphorbiaceae*

The leaves of *E. cyparissias* are used as anti-warts (Pieroni and Vandebroek, 2007). The flowers, stems, and leaves of cypress spurge are used in folk medicine in the treatment of dermatological diseases (psoriasis, eczema), respiratory diseases (asthma, bronchitis, chest congestion, throat spasm), hay fever, and against tumor development (Özbilgin et al., 2012; Stanković and Zlatić, 2014). Its seed oil is purgative. In the past, vapor of the chloroform extract was used as anesthetic agent. It is recommended for insomnia and ear constipation. Its condensed milky sap is one of the components of “emplastrum cantharides” (blister bug plaster) which is used principally to relieve deep-seated inflammation and to promote the absorption of effusions (Papp, 2004).

The whole plant has antioxidant properties given by the high amount of secondary metabolites, mainly isoprenoids (diterpenoids, triterpenoids, sesquiterpenoids—elemene and caryophyllene), phenolic acids, and flavonoids (Hemmer and Gülz, 1989; Özbilgin et al., 2012; Stanković and Zlatić, 2014). Two new diterpenoids have been identified in extracts of *E. cyparissias* (*i.e.*, cyparissin A and B) that exhibit important activity against cancerous cell lines in *in-vitro* studies (Lanzotti et al., 2015).

In Romanian ethnomedicine *E. cyparissias* is recommended for its purging, emetic (Craciun et al., 1976), antispasmodic, sedative, antifungal, anti-rheumatic, revulsive, and febrifuge effects (Milica et al., 2012) and is used for treating warts, heir loss and sciatic neuralgia (Grigore and Grigore, 2007), gastric distress, constipation, stomach pains, intestinal worms, emphysema, pleuritis, hay fever, tuberculosis, pharyngitis, mange, eczemas, arthritis, ringworm, anthrax, impetigo, nail mycosis, tooth pain, and freckles (Milica et al., 2012).

### *P. aquilinum* (L.) Kuhn—Bracken Fern, *Polypodiaceae*

Dioscorides (ca. 50 AD) in his de *Materia Medica* referred to several ferns, including *P. aquilinum*, as having medicinal values. *P. aquilinum* is thought to be a fern with potent anti-cancer properties. In India, decoctions from the rhizomes of *P. aquilinum* are drunk as an herbal tea (Baskaran et al., 2018). Dried rhizomes mixed with milk are used to relieve diabetic disorders, and tender fronds are used as vegetables (Baskaran et al., 2018). Moreover, the strong rhizomes of plant have been used directly as a food or as an ingredient of bread (by Australian, British, French, Japanese populations or by Lapp and Siberian cultures) (Vetter, 2010). It is the most common edible pteridophyte in sub-Saharan Africa, used as human food in Angola, Cameroon, DRC, Gabon, Madagascar, Nigeria, and South Africa (Maroyi, 2014).

The high concentration of phenol, flavonoid, and terpenoid compounds gives to bracken fern extracts antioxidant properties and antimicrobial activity, justifying its traditional use to treat skin diseases and gastrointestinal disorders (May, 1978; Mannan et al., 2008; Piluzza and Bullitta, 2011; Kardong et al., 2013). The presence of tannins, cardiac glycosides, anthraquinone, and cyanogenic glycosides in this species may have a negative impact on liver function (Hassan et al., 2007) and vitamin B_1_ metabolism (Fenwick, 1989; Vetter, 2010).

### *R. acetosella* L.—Red Sorrel, *Polygonaceae*

In TM the leaves of *R. acetosella* are used by the native populations of North America as treatment for warts and bruises. The aerial parts contribute to amelioration of diarrhea and stomach disorders in North America and Hungary, while seeds are used to treat diarrhea and dysentery in Hungary (Chevalier, 1996; Shale et al., 1999; Foster et al., 2000; Wegiera et al., 2007; Vasas et al., 2015). An ethnobotanical survey of medicinal plants used in Turkey revealed that leaves of *R. acetosella* are used traditionally as an analgesic and diuretic (Cakilcioglu and Turkoglu, 2010). In Iran, *R. acetosella* is widely used by traditional healers for the treatment of jaundice and fever (Amiri et al., 2014). In Poland, this species is still in use today as potherb (Łuczaj and Szymański, 2007).

The main chemical compounds in the genus *Rumex* are antraquinones (emodin, physcion, and chrysophanol in fruits and leaves, and sennoside A in fruits and roots), nepodin, and flavanoids (quercetin-3-O-glucoside) with important antioxidant activity, while the stilbenoids demonstrated to have a positive impact in cancer therapy and inflammatory diseases (Wegiera et al., 2007; Vasas et al., 2015).

In Romanian ethnomedicine *R. acetosella* is recommended for its digestive, stomachic, laxative, cleansing, antidiarrheal, cholagogue, diuretic, emmenagogue, anthelmintic, astringent, appetite-stimulant, nutritious, anti-anemic, and antiscorbutic properties (Milica et al., 2012). This plant was recommended for treating paralysis (Craciun et al., 1976), hepatitis, jaundice, cholelithiasis, constipation, pulmonary illnesses, asthma, insect bites, gout, leucorrhea, nephritis, wounds, acne, boils, ringworms, cancerous ulcerations, and fever and as mineralizing treatment and for blood purification in the spring (Milica et al., 2012).

### *V. album* L.—White Hellebore, *Melanthiaceae*

*V. album* has a long history of medicinal use, dating back as far as Hippocrates in which dilutions of the plant are prescribed to produce upward purging. Through the 1700s, preparations of *V. album* root and rhizomes were used medicinally in Europe primarily for their emetic properties (Chandler and McDougal, 2014). In the folk medicine *V. album* is used to treat rheumatism, toothache, gout, herpes, trigeminal neuralgia, and catarrh, and as an hypotensive agent (Furbee, 2009; Ujváry, 2010; Wiart, 2012; Roberts and Wink, 2013).

The medicinal, and the toxic properties of white hellebore are given by the presence of steroidal alkaloids including proveratrine, cevadine, protoveratrine, jervine, veratramine, and veratridine, the last being the most poisonous of all. Some of the symptoms of intoxication with white hellebore are hypotension, bradycardia, and paralysis (Furbee, 2009). Similar to *C. maculatum*, the alkaloids presence in all plant parts gives the species sedative and anti-inflammatory properties, antihypertensive activity being also described, especially linked to the presence of protoveratrine A and B (Krayer and Meilman, 1977).

In the Romanian ethnomedicine *V. album* is used for its neuro-sedative (Craciun et al., 1976; Parvu, 2000; Ardelean et al., 2008), hypotensive, narcotic, hypnotic, antipyretic (Parvu, 2000), anthelmintic, and insecticidal effects (Milica et al., 2012). The plant is used for treating malignant hypertension, hypertensive crisis, eclampsia, fever, eczemas, mange, itchy skin (Parvu, 2000), rheumatism, gout, eczema (Ardelean et al., 2008; Milica et al., 2012), pneumonia, whooping cough, intermittent fever, mental illnesses, psychiatric disorders, mania, mange, pruritus, psoriasis, and herpes zoster (Milica et al., 2012).

### *X. spinosum* L.—Bathurst Burr, *Asteraceae*

In ethnomedicine *X. spinosum* is used in renal disorders, for its antibacterial, antifungal, and anthelminthic properties; calming influence; wound healing properties; and the efficacy of infusions in treating benign prostate hyperplasia (Domokos et al., 2016; Aldibekova et al., 2018). It was used against rabies, to relieve chronic fevers, to abate diabetes effects, even to stimulate saliva production for its diuretic effect (Andreani et al., 2017) and “it exhibits” noticeable antioxidant activity (Aldibekova et al., 2018). In Pakistani local communities the leaves and fruits are reported to be diaphoretic, diuretic, and sedative and used for hydrophobia while the infusion of root is emetic (Aziz et al., 2018).

The main chemical compounds are polyphenols, flavones, diterpenes, sesquiterpene lactones (xanthatin), phytosterols (Domokos et al., 2016), tannins, and essential oils (Aldibekova et al., 2018). It has been showed that xanthatin can inhibit the growth of Gram positive bacteria (*Staphylococcus aureus* and *Bacillus cereus*) and fungi (*Colletotrichum gloesporoides* and *Trichothecium roseum*) (Ginesta Peris et al., 1994). *In vitro* and *in vivo* studies showed that xanthatin and xanthinosin have chemopreventive properties, inhibiting tumor cells proliferation (Ramírez-Erosa et al., 2007; Romero et al., 2015). The therapeutic properties of xanthatin can be extended also to antiviral (herpes simplex virus, feline corona virus, influenza A H_1_N_1_ and A H_3_N_2_, influenza B, repvirus, respiratory syncytial virus etc.) and anti-angiogenic activities (Romero et al., 2015).

In Romanian ethnomedicine *X. spinosum* is recommended for its anti-inflammatory, diuretic, diaphoretic, and disinfectant effects (Bojor and Pop, 2010). This plant is recommended for treating polyuria, prostatitis, selective electrolyte retention (K^+^ and Mg^2+^) in the blood serum, protection of the myocardium, prostate adenoma, nephrolithiasis (Bojor and Pop, 2010), rabies, and hyperthyroidism (Ardelean et al., 2008).

### *X. strumarium* L.—Common Cocklebur, *Asteraceae*

Although the fruit is the predominant part of *X. strumarium* used in folk medicine (Fan et al., 2019), the leaves and roots have been used for their diaphoretic, diuretic, emollient, appetite-stimulant, laxative, antirheumatic, antisyphilitic, anodyne, and sedative properties (Chopra et al., 1956). Traditionally, this species is also used as febrifuge drug and as an immunostimulant, against malaria, as well as dysentery cure, astringent, sedative, analgesic, against leucorrhea and urinary diseases, eczema and skin disease, bleeding, insect bite, to treat boils and pimples, against smallpox and stomach diseases, earache and strumous disease, leprosy, headache, and fever (Katewa, 2008; Kozuharova et al., 2019). In Yemen and Russia, *X. strumarium* is used as a medicine soothing illness, hemolytic, reducing temperature, skin diseases, antimicrobial, for ulcers, and antifungal (Aldibekova et al., 2018).

In common cocklebur the aerial parts' main compounds are the sesquiterpene lactones (guianolides, elemanolides, germacranolides, xanthinium, xanthumin, xanthatin) with important anti-inflammatory, antiviral, antitumor, and antimicrobial properties (Kamboj and Saluja, 2010; Fan et al., 2019).

A series of compounds with pharmacological importance have been identified in all parts of *X. strumarium*: glycosides (xanthostrumarin, atractyloside, carboxyatractyloside, the last being also the most toxic compound) (Kamboj and Saluja, 2010), phytosterols (xanthanol, isoxanthonol, xanthinosin, 4-oxo-bedfordia acid) (Olivaro et al., 2016), caffeoylquinic acid, thiazinodine, diacetyl xanthumin (antifungal compound), linoleic acid, and triterpenoids (botulin, betulinic acid, erythrodiol, lupeol acetate, oleanolic acid, amyrin) (Kamboj and Saluja, 2010; Yadav et al., 2015; Fan et al., 2019). In n-butanol fraction of the ethanolic extract of *X. strumarium* has been proven to possess the highest analgesic and anti-inflammatory activity (Han et al., 2007).

In Romanian ethnomedicine, the whole *X. strumarium* plant is recommended for treating diabetes (Grigore and Grigore, 2007).

## Recommended Ethnomedicinal Uses of Some Meadow Weeds in Romania and Other Neighboring East European Countries

The weed species growing on the Romanian meadows, which have to be removed by land owners or farmers if public subsidies under EU's CAP are expected, have multiple and documented uses in TM. Yet, two of these species, *C. maculatum* and *V. album* are very toxic and they are not recommended for human use, but they can be potential valuable sources of pharmaceutically active compounds.

For the majority of these meadow species have been reported details of their recommended ethnomedicinal uses in Romania (**Table 1**). On the Romanian market many commercial herbal products that have ingredients derived from the meadow weeds are on sale, including through e-commerce (**Supplementary Table 1**).

**Table 1:** Ethnomedicinal uses of the meadow weeds in Romania.

Species	Part used	Medicinal use or ailments treated	Preparation or administration	References
*Arctium lappa* L.	Leaves	Headache	Tea	(Papp et al., 2014)
	Roots	Sore throat, respiratory diseases	Tea	(Fierascu et al., 2017)
	Seeds	Cough, gastrointestinal disorders (reflux)	Tea	(Papp et al., 2011; Papp et al., 2017)
	Leaves, roots, seeds	Skin diseases (acne, dermatitis, dry scalp seborrhea, alopecia)	Decoction	(Gilca et al., 2018)
		Antimicrobial effect		(Segneanu et al., 2019)
	Roots	Expectorant, antitussive, emollient, diuretic, anti-inflammatory, digestive, renal disorders, cough, bronchitis, wounds, sores	Decoction	(Tita et al., 2009)
*Euphorbia cyparissias* L.	Aerial parts	Alopecia, dermatitis	Homeopathic remedies	(Parvu, 2000; Gilca et al., 2018)
*Eryngium campestre* L.	Rhizomes	Detoxifying, diuretic, cicatrizing, eupeptic, carminative, sedative, abdominal distention, urinary lithiasis, anorexia, gastric ulcer, convulsive cough, wounds	Decoction	(Tita et al., 2009)
*Rumex acetosella* L.	Herb	Pneumonia	Cataplasm	(Papp et al., 2011)
	Leaves	Warts, bruises	Poultice	(Butura, 1979)
*Veratrum album* L.	Roots, rhizomes	Dermatitis, scabies Anti-inflammatory, antispastic, antibacterial, hypotensive	Tincture	(Parvu, 2000; Gilca et al., 2018; Segneanu et al., 2019)
*Xanthium spinosum* L.	Aerial parts	Rabies, hyperthyroidism, prostate adenoma	Decoction	(Parvu, 2000)
*Xanthium strumarium* L.		Diuretic	2% tincture of the cocklebur germ	(Aldibekova et al., 2018)
		Bladder and urethra diseases	Adenostop^TM(*^^)^ herbal product (squeeze of the cocklebur spiny)	(Klimakhin et al., 2015)

(*) Disclaimer: Mention of proprietary products is solely for the purpose of providing specific information, and does not constitute an endorsement or a recommendation for their use.

The same meadow weeds have been used in the TM (**Table 2**) in many neighboring European Eastern countries (**Supplementary Figure 1**).

**Table 2:** Ethnomedicinal use of the meadow weeds in neighboring East European countries.

Species	Country	Part used	Medicinal use or ailments treated	Preparation or administration	References
*Arctium lappa* L.	Czech Republic	Roots	Adjuvant therapy in diabetes	Herbal tea	(European Medicine Agency, 2011)
	Estonia	Roots	Cancer treatment	Herbal tea	(Sak et al., 2014)
	Russia	Roots	Cancer treatment, antioxidant activity	Flor‐Essence™^(*^^)^ herbal tonic	(Tamayo et al., 2000)
				Infusions, decoctions, extracts or tinctures	(Spiridonov, 2008)
		Leaves	Skin wounds, dermatological disorders	Crude	(Mamedov et al., 2004)
	Ukraine	Leaves	Headache	Fresh or dried	(Sõukand and Pieroni, 2016)
		Roots	Knee ache, hair loss	Infusion	
			Blood cleansing	Tea	
	Bosnia and Herzegovina, Serbia, Kosovo	Leaves	Wound healing	Powder or boiled in milk	(Jarić et al., 2018)
	Kosovo	Aerial parts	Gastrointestinal disorders, bronchitis, lithontriptic	Decoction	(Mustafa et al., 2012)
	Bulgaria	Roots	Diuretic, ulcer	Decoction	(Leporatti and Ivancheva, 2003)
*Carduus nutans* L.	Kosovo	Inflorescence	Eczemas	Extracted with cold water for 10 days and then used as tea	(Mustafa et al., 2012)
	Turkey	Seeds (crushed)	Liver diseases	Infusion	(Bulut et al., 2017)
*Euphorbia cyparissias* L.	Kosovo	Stem	Warts	Fresh leaves topically applied	(Mustafa et al., 2012)
*Eryngium campestre* L.	Bulgaria	Roots	Diuretic, spasmolytic, prostatitic	Infusion	(Leporatti and Ivancheva, 2003)
	Turkey	Aerial and root parts	Antitussive, diuretic, appetite-stimulant, stimulant, and aphrodisiac	Infusion	(Wang, 2012)
		Root	Vulnerary	Powdered (external)	(Altundag and Ozturk, 2011)
		Leaf	Carminative, jaundice	Decoction	
		Roots	Anti-inflammatory	Crushed	(Karakaya et al., 2019)
*Pteridium aquilinum* (L.) Kuhn	Kosovo	Leaves	Anti-bacterial, diuretic	Decoction	(Mustafa et al., 2012)
*Rumex acetosella* L.	Russia	Roots	Skin wounds, dermatological disorders	Powder	(Mamedov et al., 2004)
	Turkey	Leaf	Abscess	Pounded (external)	(Altundag and Ozturk, 2011)
		Herb	Stomachic	Internal	
*Veratrum album* L.	Bulgaria	Roots	Hypotensive	Tincture	(Leporatti and Ivancheva, 2003)
	Kosovo	Leaves	Anti-lice	Decoction	(Mustafa et al., 2012)
*Xanthium strumarium* L.	Russia	Whole plant	Hepatoprotector, pulmonary and uterus cancer	Infusions, decoctions, extracts, tinctures	(Spiridonov, 2008)

(*) Disclaimer: Mention of proprietary products is solely for the purpose of providing specific information, and does not constitute an endorsement or a recommendation for their use.

From all 10 species, *A. lappa* has recorded the highest number of ethnomedicinal recommended uses, both in Romania and neighboring East European countries. On the contrary, we were not able to find any documented use of *P. aquilinum* in Romanian ethnomedicine, but in Kosovo its leaves decoction is used as antibacterial and diuretic treatment (Mustafa et al., 2012).

Medicinal plants have historically proven their value as a source of molecules with therapeutic potential, and still represent an important pool for the identification of novel drug leads. Particularly relevant examples of plant-derived natural compounds that have become indispensable for modern pharmacotherapy are the anti-cancer agents, e.g., paclitaxel and its derivatives from *Taxus* species, vincristine and vinblastine from *Catharanthus roseus* (L.) G. Don, and camptothecin and its analogs initially discovered in *Camptotheca acuminata* Decne. (Atanasov et al., 2015). There were an estimated 18.1 million new cases of cancer and 9.6 million deaths from cancer worldwide in 2018 from which in Romania alone been registered 83,461 new cases and 50,902 deaths (Ferlay et al., 2019). As a highly relevant aspect related to human health, the reviewed peer-reviewed publications reported the use of seven out of the 10 species*, i.e. A. lappa*, *C. maculatum*, *E. cyparissias*, *P. aquilinum*, *R. acetosella, X. spinosum*, and *X. strumarium*, as treatments in ethnomedicine for different types of cancer (e.g. prostatic, pulmonary, endometrial), cancerous ulcerations and tumor development in Eastern European countries, suggesting further investigations. From all the above-mentioned species, only three of them have been reported in Romanian for treatment of cancerous ulcerations (*R. acetosella*) and prostate cancer (*X. spinosum*, and *X. strumarium*). At least one commercial herbal product containing both herba Xanthii and herba Xanthii spinosi is sold on the Romanian market.

## The Raw Plant Material and Its Influence on the Authenticity of the Derived Herbal Products

The herbal products, sold as medicines or food supplements, represent a core part of the TM which has been has recognized by the World Health Organization (WHO) as a growing and expanding global phenomenon (World Health Organization, 2013). The rapidly expanding global market is expected to reach US$ 115 billion in 2020 (Raclariu et al., 2018) while the trade of medicinal plants will grow at the rate of 15%–25% annually and will reach US$ 5 trillion by 2050 (Booker et al., 2012). The increasing demand for herbals and the limited supply of many species that are harvested from the wild (Coghlan et al., 2015) is stimulating the economically-motivated adulteration (EMA) (Simmler et al., 2018). It was recently reported that 27% of almost 6,000 commercial herbal products sold in 37 countries were found to be adulterated, while in Europe almost half (47%) were adulterated. In Romania, 94% of the herbal products analyzed (n = 70) were reported to be adulterated, when their composition was compared with the labeled ingredient species (Ichim, 2019). The availability of new plant biomass while is removed from the Romanian meadows by its owners or farmers can become readily available to the interested users and could reduce the pressure on the existing sources of raw materials. Moreover, because only selected species have to be searched, identified and removed, a supplementary quality check is added to the plant raw material, before being further used or processed, which represents additional benefits for the quality of the herbal products and their benefits for the human health.

## Conclusion

The Romanian landowners or farmers have to remove from their meadows 10 plant species without fodder value or toxic to animals as a compulsory condition to receive the public subsidies under EU's CAP. For these unwanted meadow weeds have reported many and various uses in Romanian ethnomedicine, as well as in other nine neighboring East European countries. Some of the ethnopharmacological uses are highly relevant for the modern medicine, supporting the use of the removed biomass from the meadows as additional or alternative source of pharmacologically active ingredients. The local communities and the industry of herbal food supplements can take immediate advantage from these raw plant materials, readily and easily available, while assuring their environmentally-friendly, sustainable, and supportive of local traditions and commercial use.

## Author Contributions

MI conceived and supervised the research. MI and EG wrote the manuscript. MI and EG read and approved the submitted manuscript.

## Funding

This work was supported by a grant of the Ministry of Research and Innovation through Program 1–Development of the National R&D System, Subprogram 1.2–Institutional Performance–Projects for Excellence Financing in RDI, contract no. 22PFE/2018. This publication was supported by the National Core Program funded by the Romanian Ministry of Research and Innovation, project number 25N/11.02.2019, BIODIVERS 19270401.

## Conflict of Interest

The authors declare that the research was conducted in the absence of any commercial or financial relationships that could be construed as a potential conflict of interest.
