# Supplementary material for: Turning Meadow Weeds Into Valuable Species for the Romanian Ethnomedicine While Complying With the Environmentally Friendly Farming Requirements of the European Union’s Common Agricultural Policy
Source: Front Pharmacol. 2020 Apr 23;11:529. doi: 10.3389/fphar.2020.00529 (PMC7191034; doi:10.3389/fphar.2020.00529)
Supplement: Supplementary file 1 [file Image_1.pdf]

Supplementary Figure 1

Supplementary Figure 1. The geographical distribution of the East European countries where details about the ethnomedicinal uses of the meadow weeds were reported (*green color*). *The countries not included in the analysis are shaded gray.*

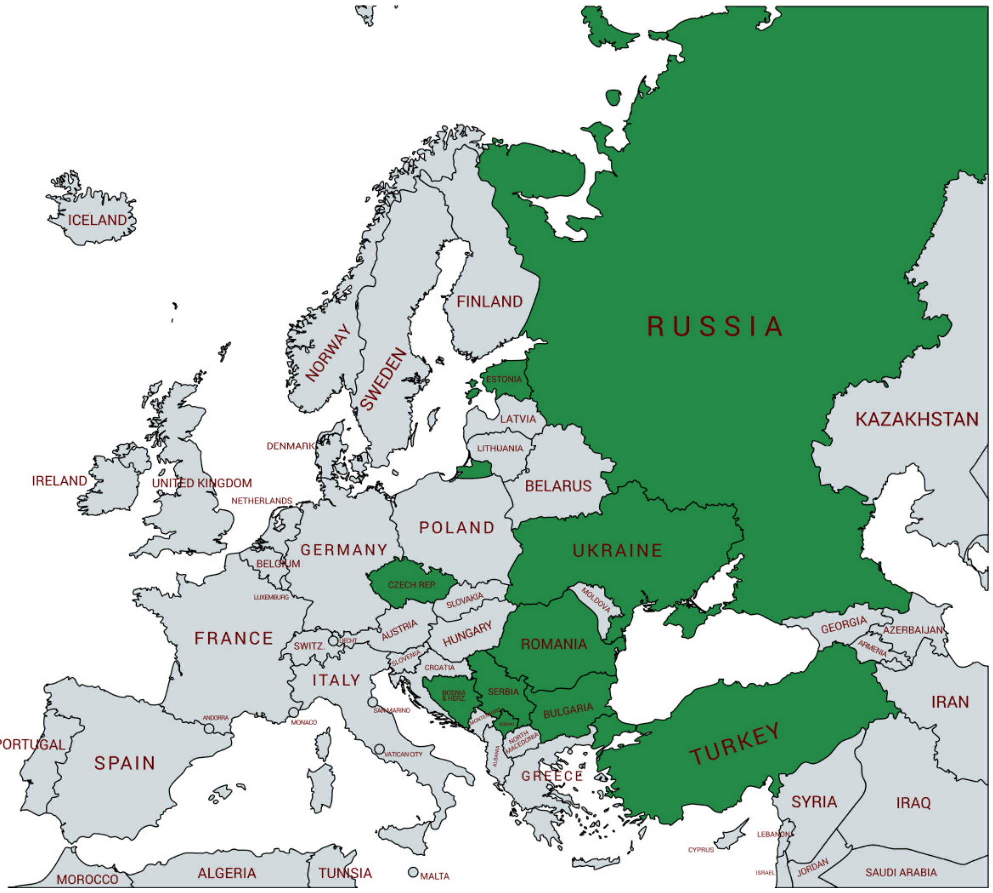

Created with mapchart.net ©

The map was created on mapchart.net
