# Supplementary material for: Turning Meadow Weeds Into Valuable Species for the Romanian Ethnomedicine While Complying With the Environmentally Friendly Farming Requirements of the European Union’s Common Agricultural Policy
Source: Front Pharmacol. 2020 Apr 23;11:529. doi: 10.3389/fphar.2020.00529 (PMC7191034; doi:10.3389/fphar.2020.00529)
Supplement: Supplementary file 2 [file Table_1.pdf]

Supplementary Table 1

Supplementary Table 1. Commercial herbal food supplements(*) sold online on the Romanian market (links accessed: 05 March 2020)

Species	Produced		Form/composition	Link
	domestic	imported		
Arctium lappa L.	X		Hydroalcoholic extract (roots)	https://www.charmecosmetics.ro/tinctura-de-brusture/
	X		Tea (roots) (+ other 9 species)	https://deprinromania.ro/ro/Ceai-Tiroido-G-20-plicuri
		X	Capsules (roots)	https://ro.coral-club.com/shop/products/91860.html
		X	Capsules (roots) (+ other 4 species)	https://www.cosmopharm.eu/produs/pachet-pentru-afectiuni-hepatice/
	X		Hidroalcoolic extract roots)	https://www.faunusplant.ro/tinctura-brusture.html
	X		Hidroalcoolic extract roots)	https://picaturi-extracte.compari.ro/dacia-plant/tinctura-de-brusture-50ml-dacia-plant-p366789743/
	X		Tea	https://stefmar-store.ro/ceai-brusture-radacina-50g
	X		Hidroalcoolic extract (flowers)	http://terrabucovina.ro/extract-brustur
	X		Capsules (+ other 7 plant species)	http://www.hofigal.eu/depurin-40-compr-6.html
		X	Capsules (roots, powder)	https://www.pcfarm.ro/produs/8168/Burdock---Brusture--
	X		Hidroalcoolic extract	https://www.pcfarm.ro/produs/7706/Tinctura-de-brusture---Dacia-Plant
		X	Powder (roots)	http://www.nutramax.ro/250-Produse-Gama-Nutra-Life-Pulberi-Bioactive-Simple/264~Protectie-Cardio-Vasculara/438-Burdock-Root---Pulbere-Bioactiva---125gr.html
		X	Tea (roots) (+ other 7 plant species)	https://www.cel.ro/ceai/ceai-din-8-plante-essiac-113gr-swanson-pMCI3MTMtNg-l/
		X	Liquid extract (+other 3 plant species)	https://www.remediu.ro/essiac-300ml-essiac-p756.html

	X		Tea (roots) (+ other 6 species)	https://www.favisan.ro/catalog.php?detalii=157
Carduus nutans L.		X	Tea (+ other 9 species)	https://bioboom.ro/ceai-anti-alergii-bio-50-g/
		X	Tea	https://oliveland.ro/product/ceai-de-ciulin-60g/
Conium maculatum L.		X	Homeopathic remedy	https://www.apoteca-farmacie.ro/conium-maculatum-ch15-1-tub-boiron--12795
Eryngium campestre L.		X	Tea (+ other 12 plant species)	https://ctscorner.ro/infuzie-de-plante/216-ceai-pentru-afectiuni-erectile-50g-8594045067873.html
	X		Syrup (aerial parts) (+ other 6 plant species	https://www.abcplant.ro/remedii/tusiflor-sirop-200-ml.html
	X		Syrup (aerial parts) (+ other 7 plant species	https://www.pfarma.ro/medicatie-pe-afectiuni/afectiuni-respiratorii/elidor-sinomax-sirop-200-ml-a013109.html
Rumex acetosella L.		X	Leaf extract (+other 3 plant species)	https://www.remediu.ro/essiac-300ml-essiac-p756.html
	X		Tea (+other 31 plant species)	https://www.drgreen.ro/produse/ceaiuri/ceai-pansament-digestiv-320g.html
	X		Extract	https://www.breslo.ro/item/extract-de-ulm-extract-de-pelin-extract-de-macris-extract-de-lamaita-687745
		X	Tea (leaves) (+ other 7 plant species)	https://www.cel.ro/ceai/ceai-din-8-plante-essiac-113gr-swanson-pMCI3MTMtNg-l/
Veratrum album L.		X	Homeopathic product	https://www.argefarm.ro/homeopate/25932-Veratrum-Album-30ch.html
Xanthium spinosum L.	X		Tea	https://www.daciaplant.ro/ceai-ghimpe.html
	X		Tea	https://www.alevia.com.ro/product/ceai-de-ghimpe/
	X		Tea	https://deprinromania.ro/ro/Ceai-de-ghimpe-U87-20-plicuri
	X		Tea	https://pentrucorpultau.ro/produs/ceai-ghimpe/
	X		Hidroalcoolic extract	https://comenzi.farmaciatei.ro/farmacie/urologie/prostata/tinctura-de-ghimpe-50-ml-dacia-plant-p305314
	X		70% hidroalcoolic extract	https://www.shopmania.ro/produse-naturiste-diverse/p-tinctura-de-ghimpe-hofigal-149215355
	X		Tea	https://www.emag.ro/ceai-

				farmanatpoieni-prostato-plant-ceai-prostata-100g-fmn00003/pd/DN23TDBBM/
	X		Tea	https://www.planteea.ro/ceai-ghimpe-50g-trident/
	X		Tea	https://stefmar-store.ro/ceai-ghimpe-iarba-50g
	X		Capsule	https://www.pcfarm.ro/produs/862/Ghimpe
	X		Capsule (+ other 5 plant species)	https://www.drgreen.ro/afectiuni/aparatul-urinar/prostatita/pufulita-cu-flori-mici-60cps-250mg-hypericum.html
	X		Gliceric extract extract 1:10, in solutiehidrogliceric a cu 50% v/v glicerinavegetala	https://www.minuneanaturii.ro/produse/prostata/extract-gliceric-ghimpe-50ml-ad-natura-pret-oferta_.html
	X		Supozitoare	http://www.elzinplant.ro/produs/Pentru%20afectiuni%20ale%20prostatei/carpizin-42
	X		Tea (+ other 6 plant species)	https://faunusplant.ro/produse/ceaiuri/ceai-retete-traditionale-prostata-180g/
Xanthium strumarium L.	X		Fruit extract (+ other plant species)	https://www.putereaplantelor.ro/adenom-de-prostata/comag-plant-b-supozitoare-elzin-plant.html?gclid=Cj0KCQjwuNbsBRC-ARIsAAzITucDN2VV51J6u05MUB3nhuZtoBbE5s-IoZGMz-ueOelX7omiv81LQmUaApXWEALw_wcB
		X	Fruit extract (+ other plant species)	https://comenzi.farmaciatei.ro/ingrijire_personala/ingrijire_corp_si_fata/tratamente/lotiune_calmanta_real_tonic_200_ml_atoclassic-p10056588
		X	Fruit extract (+ other plant species)	https://comenzi.bebetei.ro/produse-dermatologice/ingrijire-fata/creme-si-produse-ingrijire/esenta-booster-geum-sul-first-100ml-missha-p349451
	X		Fruit extract (+ other plant species)	https://www.remediu.ro/conimed-plant-supozitor-15-gr-10buc-conimed-p15887.html?utm_source=price-ro

(*)*Disclaimer*: Mention of proprietary products is solely for the purpose of providing specific information, and does not constitute an endorsement or a recommendation for their use.
